# Book Review

**Published:** 1895-12

**Authors:** 


*Dissections Illustrated.* By C. Gordon Brodie. In Four Parts. Part IV.: The Abdomen. London: Whittaker & Co. 1895.—The present number completes this excellent work, which we have favourably noticed on previous occasions. The plates showing the perineum and those showing the abdominal viscera are fully up to the previous standard, and are further illustrated by diagrams which serve the useful purpose of explaining the coloured plates. The whole work will form a valuable aid to the student in reading up anatomy in the study, and will readily recall the appearances presented by the parts seen in actual dissection.

*An Anatomical Dissertation upon the Movement of the Heart and Blood in Animals, being a Statement of the Discovery of the Circulation of the Blood.* By William Harvey, M.D., Physician-Extraordinary to King James the First; Physician-in-Ordinary to King Charles the First; and Professor of Anatomy in the Royal College of Physicians in London. Privately reproduced in facsimile from the Original Edition printed at Franckfort-on-the-Maine in the year 1628, with a Translation and Memoir, for G. Moreton, Canterbury. 1894.—This is a most interesting volume, and greatly to be prized by the bibliophile and physician. A facsimile reproduction by collotype of the original work printed for Harvey at Frankfort in 1628, with ample margins, rough edges, and parchment-board binding, the *format* is indeed delightful. An anonymous, but apparently new, translation of the text follows the main body of the work, and preceding it is a sketch of the life of the illustrious man, while the portrait of Harvey in the National Portrait Gallery reproduced in photogravure serves for the frontispiece. It is to be regretted that the College of Physicians refused to allow their probably better portrait of their ever famous Fellow to be reproduced for this work. While hardly perhaps rivalling as a medical classic the translation of Harvey's works by Dr. Willis, published nearly fifty years ago by the Sydenham Society, this is distinctly a most precious possession.

*The Lumleian Lectures on certain points in the Ætiology of Disease,* delivered before the Royal College of Physicians, 1892, to which is added the Harveian Oration delivered before the College in 1893, with a Biographical Notice of Harvey, and an Appendix of Statistical Tables. By P. H. Pye-Smith, M.D., F.R.S. Pp. 236. London: J. & A. Churchill. 1895.—The report of the Lumleian lectures contained in this volume has the portions which on the occasion of their delivery were omitted on account of want of time. The biographical notice of Harvey, which here follows the Harveian Oration, is from the last edition of the *Encyclopædia Britannica.* The whole volume should be read by those who have not already done so, but it is not a book to epitomise or condense.

*Diabetes, and its Treatment.* By A. Vintras, M.D. London: Baillière, Tindall and Cox. 1895.—This little book is made up of twenty-four pages of matter on diabetes and forty-two pages of catalogue of the publishers. The latter we are not called upon to review, and the former is not worth it. The author in the preface alludes to "this pathological study of diabetes," but we find no reference to pathology afterwards; and he states that he has given "due prominence to the practical questions of diagnostic, prognostic, and to special therapeutical indications," whereas it appears to us that he has given little prominence to any of the numerous interesting features of the disease. His diagnostic summary is as follows: "Any person who feels the necessity of drinking large quantities of liquid, who is easily fatigued, who becomes thin without any apparent cause, whose sight becomes so weak as not to allow him to read without powerful glasses, should be suspected of diabetes, and his urine should be examined." Surely it was not necessary to write a book to tell the profession this, and is it credible that the author does not examine the urine in cases not included in the above description? It has always appeared to us that no examination of any patient is complete without an examination of the urine for both albumen and sugar. We can find no *raison d'être* for such a book as this, and cannot congratulate either the author or the publishers on its appearance. Many good books on diabetes are within easy access; a perusal of this one is a mere waste of the small amount of time which it requires.

*Considérations sur les Ruptures de l'Utérus et du Vagin pendant l'Accouchement et la Valeur de la Laparotomie dans leur Traitement.* Par le Docteur G. I. Andronesco. Pp. 22. Bucarest: Imprimerie de L' "Indépendance Roumaine." 1895.—This is a small pamphlet treating of thirty-five cases of rupture of the uterus and vagina. A brief outline is given of each case, with an account of the *post-mortem* examination when one was performed. After delivery of the fœtus by forceps, two cases were subjected to laparotomy and the rents sewn up. Both cases recovered. The success attending these operations was such that the author thinks that laparotomy is the operation of the future in these cases. Of the thirty-five ruptures recorded only fourteen recovered. Various statistical tables of the cases are given, and the book concludes with an account of the methods adopted at the Bucharest Lying-in Hospital. The work is illustrated by four woodcuts, and is well worth perusal.

*The Extra Pharmacopœia.* By WILLIAM MARTINDALE and W. WYNN WESTCOTT, M.B. Eighth Edition. Pp. xxviii., 581. London: H. K. LEWIS. 1895.—This little book is of great value to the practitioner. The new edition has the same features which have made the former ones so generally popular, and the information contained in it can be relied on as being thoroughly up to date. One hundred and ten pages of new matter have been added.

*A New Method of Inhalation for the Treatment of Diseases of the Lungs.* By W. H. SPENCER, M.D. Pp. 54. London: THE SCIENTIFIC PRESS, LIMITED. 1895.—The principles and conditions of this method of treatment, and the vaporiser as a means to its practical application, are here fully described; and "of all the methods devised for the direct application of remedies to diseased parts in the interior of the lungs, that commonly known as inhalation has been the most popular," but it has also been the most barren of results. Dr. Spencer sets himself to enquire where the principle of the method has been at fault, and whether it may not be made to give better results. His vaporiser consists of a stout wire frame attached to a circular and firm wooden stand. Two pans are suspended on the frame over the source of heat. One of these pans contains the liquid to be vaporised, the other contains water; the evaporating pan is therefore immersed in a water bath, and has an oil lamp underneath. It will work for twenty-four hours without needing renewal of the water, the volatile oils being supplied from time to time as required. By the use of this apparatus the patient is placed in a medicated atmosphere, and is kept in it, always, if needs be, or by night only, as may be convenient. Various more or less potent antiseptic oils are supplied for use in different diseases, and we have found them to be of much service in cases of whooping cough and of phthisis.

*A Book of Detachable Diet Lists.* Compiled by JEROME B. THOMAS, M.D. Philadelphia: W. B. SAUNDERS. 1895.—The busy practitioner has little time to write out systems of diet for the individual patient, but this set of ten lists will include all the essential varieties of diet table. These lists are decidedly American, and will need a few erasures for use in this country.

*The Treatment and Education of Mentally Feeble Children.* By FLETCHER BEACH, M.B. Pp. 32. London: J. & A. CHURCHILL. 1895.—Dr. Beach tells us he has laboured twenty years among feeble-minded children, so that when to "summarise his observations" he produces a book which with the title-page and preface consists of only 32 pages, of well-leaded pica type, we are reminded of the parturient mountain and the birth of the mouse. In so small a pamphlet—for it really is nothing more—no one could expect to find more than the barest outlines of the treatment and education of mentally-feeble children.

*Clinical Lectures on the Prevention of Consumption.* By WILLIAM MURRELL, M.D. Pp. viii., 103. London: BAILLIÈRE, TINDALL AND COX. 1895.—In these lectures, originally delivered at the Westminster Hospital and published in the *Medical Press*, Dr. Murrell has brought together a concise and suggestive collection of facts dealing with the preventive aspect of tubercular disease. We trust that, in this more permanent form, his lectures will have a wider career of usefulness.

*Medical Handbook of Life Assurance.* By JAMES EDWARD POLLOCK, M.D., and JAMES CHISHOLM. Fourth Edition. Pp. 214. London: CASSELL AND COMPANY, LIMITED. 1895.—This work has been thoroughly revised and brought down to date. Some such manual is necessary for the guidance of medical examiners, in order that there may be something like uniformity in the rating of second-class lives. There is no difficulty in reporting on the entirely eligible, nor is there in excluding the entirely ineligible, but the intermediate cases are often difficult to deal with. We have learnt to be exceedingly suspicious of those candidates for insurance who have forgotten all details relating to the cause of death of the various members of the family, and the certificates of death obtained from Somerset House or the local registrars will often protect the office against the acceptance of an altogether unjustifiable risk.

*The Johns Hopkins Hospital Reports.* Vol. IV., No. 6. *Report in Surgery*, II. Baltimore: THE JOHNS HOPKINS PRESS. 1894.—This report contains the results of operations for the cure of cancer of the breast, performed at the Johns Hopkins Hospital from June, 1889, to January, 1894, by Dr. William S. Halsted. The operation advocated and practised by Dr. Halsted is a most radical one, involving removal not only of the whole mammary gland with its overlying skin, but also the whole contents of the axilla, as well as the greater part of the pectoralis major and minor muscles with their fascial sheaths. Great importance is attributed to the removal of all this tissue in one piece, so as not to divide lymphatic vessels containing cancer cells; and with this view, nothing but clean incisions are permitted, all tearing and scraping being strictly prohibited. Fifty cases are recorded, with only three local recurrences, comparing very favourably with the results obtained by other operators who have adopted a less radical operation. This volume furnishes additional evidence of the good work being done at the Johns Hopkins Hospital, and should be studied with care by all operating surgeons.

*Saint Bartholomew's Hospital Reports.* Vol. XXX. London: Smith, Elder, & Co. 1894.—St. Bartholomew's Hospital Reports are always interesting. This volume opens with an account by Dr. Gee of a very rare form of disease—an aneurysm of the ascending part of the arch of the aorta taking the natural position of the heart. In the previous volume Mr. Butlin related how he had tried to do with only a modification of strict antiseptic measures in surgery, and how his results had consequently fallen short of what might be expected from aseptic methods; in the present volume he tells us how good his results have been with strict asepsis. In some cases his wounds suppurated, but as house-surgeons and dressers became gradually initiated into the details of the method the frequency of suppuration decreased. Mr. Thomas Smith reports a case in which the superficial femoral, the deep femoral, and the popliteal arteries with their corresponding veins were ligatured in the course of the removal of a large sarcomatous tumour of the thigh, without damage to the vitality of the limb; but the veins had become occluded and the lumen of the artery very much diminished by pressure before the operation, so that no doubt the collateral circulation had become partly established. The great omentum is the subject of a paper by Mr. McAdam Eccles, and from a perusal of the paper one finds there is much of interest to be said about its anatomy, physiology, and pathology; and perhaps the most interesting question discussed is, as to the cause of the symptoms when, in cases of strangulated hernia, only the omentum is found nipped in the sac. We cannot say any clear light is thrown on this point, but the various views on the question are ably discussed. We can only refer to a few among the many contributions, not because we consider them the most valuable, but because space only allows us to mention those in which we have felt the most interest.

*Transactions of the Epidemiological Society of London.* New Series. Vol. XIII. London: Shaw and Sons. 1894.—This volume, in no way inferior to preceding volumes of the Series either in variety or in the individual interest of the contributions, contains nine papers, ranging from "The History of Epidemiology in England," by Dr. J. F. Payne, to "Cholera in Persia," by Dr. Camposampiero. All are of the most interesting nature and will repay careful perusal. Not the least interesting are Dr. Payne's address and Dr. Clemow's contribution on "Cholera in Russia." As a supplement, we have for the first time in print a contemporary treatise on the Great Plague in 1666, written by William Boghurst, an apothecary in St. Giles'-in-the Fields, then thirty-four years of age. The manuscript of this interesting work, which has been for a long time in the British Museum, consists of eighty-five leaves, small quarto. It is most curious reading from start to finish, and shows how the medical mind of those days was hopelessly groping about in the dark for the truth. For instance, we are given twenty-one

"diagnosticks," one hundred "evil signes or presagers of the plague," all of the most remarkable nature. It seems, in fact, as if any symptom complained of by a patient was put down as a sign or "diagnostick," and that our author was an acute observer. Of amulets, &c., he has nothing to say "more then what the prophet Jonas saith, 'They that believe lying vanities forsake their own mercies'"; but he gives a long list of things to be avoided, of which we may mention all sins, "Lust, Pride, & whoredom, wantonness, and prophaneness," as typical examples of the firm belief in the plague being the visitation of the Almighty. Boghurst does not believe in bleeding, but recommends issues for fat people "if they run well." His pharmacopœia was most extensive and peculiar, and contains many drugs whose value is not now appreciated. Dr. Payne, who has written an introduction to the treatise, says that it contains the best medical account of the great epidemic which has been preserved.

*Transactions of the Royal Academy of Medicine in Ireland.* Vol. XII. Dublin: Fannin and Company. 1894.—This volume contains no less than forty-five original papers on a great variety of topics: many of them are well-illustrated reports of original work, and others give us the latest views on theoretical questions in pathology. The weekly journals have given us summaries from time to time, but the collected series of papers forms an interesting volume, which will amply repay the expenditure of the time needful for a careful review. On page 65 we find an instructive summary of the contrast between exophthalmic goitre and myxœdema, and a comment on the beneficial effects of partial thyroidectomy.

*Annual Report of the Department of Health of the City of Chicago for 1894.* 1895.—The city of Chicago contains somewhat over a million and a half (census, 1,567,727) persons, composed of many nationalities, and with a large unsettled or floating population. This city was, in the spring of 1893, invaded by small-pox—an experience not unfamiliar in Chicago, and fraught with recollections of previous disaster. Thus, in the 1863–65 epidemic the disease so persisted and continued to spread that the deaths in February and March, 1865, in the third year of its prevalence, exceeded those in the earlier period of the epidemic; in the 1871–75 epidemic, extension of the disease continued over forty-seven months, and in the 1880-83 epidemic the disease persisted for thirty-nine months. In contradistinction to these, the epidemic of 1893–94, although for ten months it progressed and had already assumed formidable proportions, leading to 157 deaths in May, 1894, had its progress checked by very special action during that month; so that in June there was a reduction of more than one-third of the number of cases, and in July the epidemic as such "was substantially under control, and thenceforth steadily declined, until the disease lost all significance as a public health factor." At this time Chicago was fully ripe for an epidemic of small-pox equal to or exceeding that of any previous epidemic. Vaccination had been gravely neglected for many years. Fifty-nine per cent. of all cases received and treated were found to be unvaccinated, revealing a worse condition as to vaccination than in the epidemic of 1881-83, the most serious, most prolonged, and most fatal of all its epidemics. As might be expected in cosmopolitan Chicago, several districts are so distinctly foreign as to necessitate the employment of interpreters to work in them, and in these districts particularly the disease flourished and spread. The people resisted every effort of the Department to stamp out the disease; they refused to be vaccinated; they formed mobs to resist the isolation of cases. They secreted cases whenever and wherever they could; sometimes the house was barricaded against the inspectors. On one occasion an irate father, revolver in hand, stood by the bedside of his child and dared any official of the municipality to come within five feet of his offspring; one husband, when he learned that his wife and children's unvaccinated condition was the cause of his trouble, wished to kill the wife at once. In such case and under such conditions—conditions presenting no ordinary difficulties—it became obvious that prompt and radical measures alone could be of any avail, and the authorities, having determined upon the suppression of the outbreak, set about it in a whole-hearted and complete manner which does them infinite credit. To quote Dr. Reynolds, the Commissioner of Health: "This suppression was effected by an achievement of preventive medicine, . . . unparalleled as yet in sanitary administration, to-wit, the wholesale vaccination of a population of sixteen hundred thousand people, among whom more than a million free vaccinations were performed—one-third within thirty days after authority was finally secured from the City Council for the employment of men and the purchase of material,—586,500 [or more than half a million vaccinations, covering a population of more than twice the size of Bristol] in a single month. By the close of June small-pox was at an end as an epidemic disease . . . 'a disease of unusual and increasing prevalence at the time.'" In spite of the many and serious difficulties inherent in such a situation, so great was the tact, forbearance, and courage shown by the five hundred special vaccinating officers, who had to face not only abuse, but also personal violence, that, without resort to force, and without precipitating riot or bloodshed, such as occurred elsewhere, in all but four cases vaccination or removal was peaceably carried out. The planning of such a campaign was an effort of genius, its successful execution a masterpiece of executive ability unequalled in preventive medicine, and to remain unequalled, we fear, in the Old World, with its growing tenderness for private fads, its hesitancy to oppose vulgar prejudice, and its half-heartedness in municipal control in matters not understanded of the people. Chicago may well be congratulated, not only on the New World pluck which suggested this beneficent policy in the face of extreme odds, but also on the New World enterprise which brought it to a successful issue, and on the signal and gratifying success in the conservation of life which has resulted. The following table, giving the particulars relating to the 2,332 cases in hospital, shows well the severity of the type of disease; the case mortality amongst all classes being as high as 27.7, while that amongst the unvaccinated at all ages was as high as 36.2 per cent:

| Age. | Whole Number Treated. | Number Vaccinated. | Number of Vaccinated that Died. | Mortality per cent. | Number not Vaccinated. | Number of not Vaccinated that Died. | Mortality per cent. | Mortality per cent. both classes. |
|---|---|---|---|---|---|---|---|---|
| From 1—6 ... | 648 | 64 | 8 | 12.5 | 584 | 257 | 44 | 40.89 |
| From 7—15 ... | 259 | 80 | 3 | 3.75 | 179 | 41 | 22.9 | 16.98 |
| From 15—25 ... | 469 | 251 | 45 | 17.92 | 218 | 74 | 33.94 | 25.37 |
| From 25—35 ... | 542 | 313 | 54 | 17.28 | 229 | 74 | 23.64 | 23.8 |
| Over 35 ... | 414 | 285 | 51 | 17.89 | 129 | 39 | 30.23 | 21.73 |
| Totals and percentages ... | 2332 | 993 | 161 | 16.21 | 1339 | 485 | 36.22 | 27.7 |

In the recent epidemic in Bristol, the case of mortality amongst all classes did not exceed 10 per cent.

*Vocabulary of Diseases.* Prepared by J. G. KERR. Pp. 35. Shanghai: AMERICAN PRESBYTERIAN MISSION PRESS. 1894.—This is a dictionary of Chinese medical terms, based on Thomson's vocabulary and Whitney's anatomical terms. It has been prepared for the Committee on Nomenclature of the Medical Missionary Association of China, and must be very useful to those whom it may concern.

*Year-book of the Scientific and Learned Societies of Great Britain and Ireland.* London: CHARLES GRIFFIN AND COMPANY, LIMITED. 1895.—This, the twelfth annual issue, is a more complete chronicle than its predecessors, but we notice several inaccuracies in the reports of local societies. It looks as if the various Secretaries had not had a chance of revising their proofs.

*Burdett's Hospital and Charities Annual.* 1895. By HENRY C. BURDETT. London: THE SCIENTIFIC PRESS (LIMITED).—*Burdett's Hospital Annual* has become a necessity for all who are interested in the question of hospital management or who require statistics in any subject connected with medical charities. When Mr. Burdett confines h s attention to the collection of facts and statistics, no work could be done more satisfactorily, but when, in reference to hospital administration and the medical profession, he enlarges on the reforms which he considers necessary and even urgent, he is treading on more debatable ground, and he must not be surprised if we say that his opinions materially detract from the value of the book. We notice that the errors in the last two numbers which we pointed out have been rectified.

*Hermes Medical Supplement.* Vol. I., No. 1. Aug. 20th, 1895. Sydney: GEO. ROBERTSON AND COMPANY.—This small contribution to medical literature, the Journal of the Sydney University Medical Society, is to be published once in each term. We wish the infant a vigorous and continued growth, such as may be expected from the combination of the talents of graduates and undergraduates of the Sydney University.

---

We are glad to notice that with the new year Mr. W. Saunders, of Philadelphia, is to issue an *American Year-Book of Medicine and Surgery.* It is to be edited by Dr. George M. Gould, the editor of the *Medical News.* This is a guarantee that its summaries will be crisp and informing.

Dr. Unna's *Histopathology of the Diseases of the Skin*, translated by Dr. Norman Walker, is announced by Mr. William F. Clay, of Edinburgh, as nearly ready. This English edition is being revised and added to by the author, and will have several illustrations which have been specially drawn for it.

---

